# Abnormal scaffold attachment factor 1 expression and localization in spinocerebellar ataxias and Huntington’s chorea

**DOI:** 10.1111/bpa.12872

**Published:** 2020-07-13

**Authors:** Nicola Buckner, Kevin C. Kemp, Helen L. Scott, Gongyu Shi, Caroline Rivers, Andriana Gialeli, Liang-Fong Wong, Oscar Cordero-LLana, Nicholas Allen, Alastair Wilkins, James B. Uney

**Affiliations:** ^1^ Bristol Medical School Translational Health Sciences University of Bristol Bristol UK; ^2^ Institute of Clinical Neurosciences Bristol Medical School Translational Health Sciences University of Bristol Bristol UK; ^3^ School of Biosciences Cardiff University Cardiff UK

**Keywords:** Huntington’s chorea, Parkinson’s disease, polyglutamine, RNA binding protein (RBP), SAFB1, spinocerebellar ataxia

## Abstract

SAFB1 is a DNA and RNA binding protein that is highly expressed in the cerebellum and hippocampus and is involved in the processing of coding and non‐coding RNAs, splicing and dendritic function. We analyzed SAFB1 expression in the post‐mortem brain tissue of spinocerebellar ataxia (SCA), Huntington’s disease (HD), Multiple sclerosis (MS), Parkinson’s disease patients and controls. In SCA cases, the expression of SAFB1 in the nucleus was increased and there was abnormal and extensive expression in the cytoplasm where it co‐localized with the markers of Purkinje cell injury. Significantly, no SAFB1 expression was found in the cerebellar neurons of the dentate nucleus in control or MS patients; however, in SCA patients, SAFB1 expression was increased significantly in both the nucleus and cytoplasm of dentate neurons. In HD, we found that SAFB1 expression was increased in the nucleus and cytoplasm of striatal neurons; however, there was no SAFB1 staining in the striatal neurons of controls. In PD substantia nigra, we did not see any changes in neuronal SAFB1 expression. iCLIP analysis found that SAFB1 crosslink sites within ATXN1 RNA were adjacent to the start and within the glutamine repeat sequence. Further investigation found increased binding of SAFB1 to pathogenic ATXN1‐85Q mRNA. These novel data strongly suggest SAFB1 contributes to the etiology of SCA and Huntington’s chorea and that it may be a pathological marker of polyglutamine repeat expansion diseases.

## Introduction

RNA binding proteins (RBPs) play an important regulatory role in cellular metabolism by defining the complex interactions of coding, structural and regulatory RNA species (23). Recent studies have found that mutations in specific RBPs cause amyotrophic lateral sclerosis (ALS) and some forms of ataxia (4, 17). As neurons are post‐mitotic and have highly complex transcriptomes, they will be particularly susceptible to the changes in gene regulation brought about by altered RBP function. This hypothesis is supported by recent findings suggesting that altered RBP function contributes to the death of neurons seen in polyglutamine (polyQ) expansion diseases (e.g. spinocerebellar ataxia type 1 (SCA1) and Huntington’s disease (HD)) (4, 25). Historic evidence suggested that HD was caused by the mutant huntingtin protein (mHTT) losing function and due to the formation of neurotoxic fragments of mHTT (5). However, the sequestration of RBPs by the translated mutant proteins has also been shown to contribute to pathology due to a loss and/or toxic gain of RBP function (reviewed by (9, 37). Proteins containing a pathological number of polyglutamine repeats (e.g. huntingtin and ataxin‐1) are reported to lose normal functions and/or gain toxic functions due to the aberrant binding of proteins involved in the control of transcription, splicing and RNA processing (9, 22). Expansions in polyglutamine and hexanucleotide repeat regions have recently been shown to be translated by repeat‐associated non‐ATG translation (RAN translation). RAN translation produces chains of dipeptide protein that are bound by RBPs and further implicates alerted RBP function in disease pathology (6, 19, 26, 34). Furthermore, mutations in the RBPs, TDP‐43 (14), FUS (43) and MATR3 (13) have also been found to cause ALS directly linking altered RBP function in the etiology of human neurodegenerative illnesses.

Scaffold attachment factor B1 (SAFB1) is a DNA and RNA binding protein first identified on the basis of its ability to bind scaffold attachment regions (27) and as a repressor of the Hsp27 promoter DNA elements (30). Subsequent to these findings, its role as a tumor suppressor was investigated (29) and it was found to play a role in DNA repair (2). More recently the neuronal functions of SAFB1 were investigated and it was found to be expressed in the CNS with expression levels being particularly high in the hippocampus and cerebellum (28). In addition, analyses of SAFB1’s neuronal RNA binding partners showed that SAFB1 bound primarily within exon sequences, 3′UTRs and with miRNAs and long non‐coding RNAs (35). This work also showed that SAFB1 bound purine‐rich motifs to regulate the splicing of neuronal genes and that it regulated dendritic spine formation (35). SAFB1 was also recently reported to be required for FUS to regulate splicing (46) and to interact with the LC‐3 conjugation machinery to load extracellular vesicles (EVs)(21). As SAFB1 shares properties with RBPs known to be implicated in human neurodegenerative disease, is expressed at high levels in the cerebellum, translocates to nuclear stress bodies (nSBs) following the exposure of cells to a variety of stresses and binds *ATXN1* we investigated its expression in SCAs alongside other neurodegenerative diseases.

## Materials and methods

### Donor tissue

Post‐mortem formalin‐fixed paraffin‐embedded (FFPE) sections from patients diagnosed with SCA, HD, PD and control cases with no history of neurological disease were obtained from the MRC London Neurodegenerative Diseases Brain Bank. Donor tissue was collected via a prospective donor scheme and utilized in this study under ethics approval number LNDBB1778. Cerebellar tissue sections from patients diagnosed with multiple sclerosis were obtained from the Multiple Sclerosis Tissue Bank, Imperial College, London, UK and utilized in this study under research ethics committee approval 08/MRE09/31. Post‐mortem donor brain tissue was fixed in neutral buffered formalin and embedded in paraffin. Tissue sections were cut to between 7 and 10 µm and mounted onto glass slides. Details of all patient cases are given in Table 1.

**Table 1:** Clinicopathological details of donor cases used in this study.

Case	Brain region	Pathological diagnosis	Age (years)	PMD (h)	Sex (M/F)	Cause of death
1	Cerebellum	Control	74	66	F	Pneumonia
2	Cerebellum	Control	90	45	M	Myocardial infarction
3	Cerebellum	Control	66	78	F	Metastatic colorectal cancer
4	Cerebellum	Control	65	26	M	Metastatic prostate cancer
		Mean	73.8	53.8	50%	
		SEM	5.8	11.5	F	
5	Cerebellum	SCA1	76	24	M	Cancer (unknown primary)
6	Cerebellum	SCA	54	31.5	F	Not available
7	Cerebellum	SCA	68	48	M	Not available
8	Cerebellum	SCA	74	33	F	Not available
9	Cerebellum	SCA	86	Not available	M	Bronchopneumonia
		Mean	71.6	34.1	60%	
		SEM	5.3	5	F	
10	Striatum	Control	51	33	F	Lung cancer
11	Striatum	Control	63	23	M	Colon cancer
12	Striatum	Control	89	43	F	Cancer
13	Striatum	Control	85	45	M	Urethral cancer
14	Striatum	Control	65	26	M	Metastatic prostate cancer
15	Striatum	Control	82	47	M	Congestive cardiac failure
16	Striatum	Control	84	34	F	Sepsis; metastatic breast cancer
		Mean	74.1	35.9	43%	
		SEM	5.4	3.6	F	
17	Striatum	HD (grade 2)	83	55	F	Myocardial infarction
18	Striatum	HD (grade 2)	79	26	M	Huntington’s disease
19	Striatum	HD (grade 3)	54	19	M	Huntington’s disease
20	Striatum	HD (grade 3)	57	66	F	Bronchopneumonia
21	Striatum	HD (grade 3)	83	24	F	Not available
22	Striatum	HD (grade 4)	64	47	M	Not available
23	Striatum	HD (grade 4)	55	60	F	Not available
24	Striatum	HD (grade 4)	79	45	F	Acute myocardial infarction
		Mean	69.3	42.8	63%	
		SEM	4.6	6.3	F	

### Immunohistochemical staining of human brain tissue

Formalin‐fixed paraffin‐embedded (FFPE) sections were immunostained with rabbit anti‐SAFB1 antibody (1:50) (A300‐811A, Bethyl Laboratories, UK), or mouse anti‐polyglutamine antibody (1:1000) (1C2, Millipore). Sections were deparaffinized in Histo‐clear and rehydrated in graded alcohol solutions. Antigen retrieval was carried out by microwaving slides immersed in 0.1mM sodium citrate buffer (pH 6) with 0.05% (v/v) Tween 20 until boiling. Slides were left to stand for 5 minutes, re‐heated again and boiled for 1 min, left to cool in buffer for 20 minutes and then transferred to running tap water. Endogenous peroxidases were neutralized by incubating sections for 15 minutes in 1% hydrogen peroxide diluted in distilled water. Immunohistochemical staining was carried out using an avidin–biotin complex staining kit (VECTASTAIN Elite ABC HRP Kit (Peroxidase, Universal), PK‐6200, Vector Laboratories). After washing in phosphate‐buffered saline (PBS pH 7.4) with 0.05% (v/v) Triton X‐100, non‐specific binding was blocked using normal horse serum (VECTASTAIN blocking solution) diluted 1:100 in PBS‐T for 45 minutes. Sections were incubated with primary antibody diluted in blocking solution overnight at 4°C. Sections incubated in antibody diluent only served as no primary antibody controls. Sections were washed in PBS, then incubated in secondary antibody (VECTASTAIN biotinylated horse anti‐rabbit/mouse, 1:50) diluted in VECTASTAIN blocking solution. Following PBS washes, sections were incubated in ABC reagent (VECTASTAIN Elite ABC Reagent, 1:50) and antibody binding was visualized using 3,3′‐diaminobenzidine (DAB) (Vectorlabs DAB substrate kit, no nickel). After washing in distilled water, nuclei were visualized by counterstaining in Ehrlich’s hematoxylin, before rinsing slides in running tap water. Sections were dehydrated in graded alcohol solutions, cleared in xylene and mounted under glass coverslips using DPX mountant. Image capture was carried out using a Nikon eclipse i80 microscope with Nikon Ci‐SMS camera, and Image‐Pro plus software, or using an Aperio Scanscope CS2 digital slide scanner (Leica) and Leica ImageScope software.

### Immunofluorescent labeling of human brain tissue

Slides were dewaxed in Clearene, immersed in 100% alcohol then washed in running tap water for 10 minutes. Antigen retrieval was carried out as described for immunohistochemical staining. Slides were washed in running tap water, then washed in PBS (pH 7.4). Protein block was carried by incubating with protein blocking solution (10% normal goat serum diluted in PBS with 0.01% Triton X‐100) for 1 hour at room temperature. Sections were double immunolabeled with rabbit anti‐SAFB1 (1:200) (A300‐811A, Bethyl Laboratories) and either mouse anti‐calbindin‐D‐28K (1:500) (Sigma‐Aldrich, UK) or mouse anti‐SMI‐34 (hyperphosphorylated neurofilament, 1:500) (Covance, US). Sections were incubated in primary antibodies diluted in blocking solution overnight at 4°C. Sections were washed in PBS before incubation with fluorescently conjugated secondary antibodies diluted in blocking solution for 1 hour at room temperature, protected from light (Alexa Fluor 488 goat anti‐rabbit (1:500) or Alexa Fluor 555 goat anti‐mouse (1:500) (Invitrogen, UK)). Sections were washed in PBS, then incubated with DAPI (1 µg/mL in PBS) (Thermo Fisher Scientific, UK) for 5 minutes. Following a final PBS wash, sections were mounted in 50% PBS/50% glycerol under glass coverslips sealed with clear nail varnish. Slides were stored at 4°C in the dark prior to imaging using a Nikon C1 confocal microscope with EZ viewer software.

### Analysis of SAFB1 immunohistochemistry in cerebellar Purkinje cells

Prior to all immunohistochemical analyses, sections were coded and the investigator blinded to their identity. The analysis of Purkinje cells was based on our previous method (15). To analyze DAB immunolabeling of SAFB1 in Purkinje cells, cerebellar sections were scanned using an Aperio Scanscope digital slide scanner. The Purkinje cell layer was traced and the distance measured using annotation tools within the Aperio ImageScope software. Purkinje cells were identified by their characteristic morphology and localization within the cerebellar sections. The perikaryon of each Purkinje cell that had a clearly visible nucleus was traced and the area in µm^2^ of each perikaryon measured. Each Purkinje cell was categorized as either positive (brown staining) or negative (no brown staining) for SAFB1 immunoreactivity within the nucleus and cytoplasm.

### Analysis of SAFB1 immunohistochemistry in cerebellar white matter neurons

To analyze DAB immunolabeling of SAFB1 in large neurons within cerebellar white matter, the dentate nucleus was identified at low microscopic magnification as a characteristic grey matter ribbon running through the cerebellar white matter. Images were taken at regular intervals along the whole of the dentate nucleus tissue tract within each section. A minimum of 15 images was acquired per section. Large principle neurons were identified within each image by their characteristic size and morphology and graded as either positive or negative for SAFB1 immunoreactivity within the nucleus and cytoplasm.

### Analysis of SAFB1 immunohistochemistry in striatum

To analyze DAB immunolabeling of SAFB1 in the striatum, images were captured at 20x magnification from across the whole stained striatal section. A minimum of 15 images was analyzed per section. The number of cells containing SAFB1 immunoreactivity in the nucleus, cytoplasm, or both, were counted manually using ImageJ software. For polyglutamine (1C2) staining, the number of cells positive for immunopositivity in either the nucleus or cytoplasm was counted manually using Image J software.

### Individual‐nucleotide resolution UV crosslinking and immunoprecipitation (iCLIP)

To identify the RNA binding partners of SAFB1 iCLIP experiments in triplicate were performed as previously described (1, 35). The filtered iCLIP tag sequences were mapped to the human genome sequence (version hg19/GRCh37) allowing one mismatch using Bowtie version 1.0.1. Comparing cross‐link nucleotide positions from three independent replicates assessed reproducibility and allowed the Z‐score analysis of enriched pentamers at cross‐link nucleotides to be calculated (1, 35).

### Transfection of HeLa cells with ATXN1 plasmids

HeLa cells grown in high‐glucose DMEM (supplemented with 10% v/v fetal bovine serum and 10 mM l‐glutamine) were plated at a density of 900 000 cells/10 cm culture dish. After 24 h, HeLa cells were transfected with DNA plasmids containing FLAG‐tagged ataxin1 (ATXN1) with either a normal CAG repeat tract (pcDNA1 Flag ATXN1[30Q]) or a pathologically expanded CAG repeat tract (pcDNA1 Flag ATXN1[85Q]). pcDNA1 Flag ATXN1[30Q] and pcDNA1 Flag ATXN1[85Q] were kind gifts from Huda Zoghbi (41) (Addgene plasmid #33236 and #33237, respectively). Transfections were carried out in Opti‐MEM reduced serum media (Thermo Fisher Scientific), using FuGENE 6 (Promega) transfection reagent according to the manufacturer’s instructions. A full media change was carried out 6 hours after transfection and cells were left for 48 hours before use in RNA immunoprecipitation experiments.

### RNA immunoprecipitation

All stages of the RNA immunoprecipitation procedure were carried out in RNase‐free conditions. HeLa cells were washed in ice‐cold PBS and scraped on ice in 1ml of lysis buffer containing protease inhibitors and RNAse inhibitors. Lysates were centrifuged and the supernatant pre‐cleared by incubating with protein G magnetic beads (Dynabeads, Thermo Fisher Scientific) washed in lysis buffer. Pre‐cleared protein lysates were quantified using a bicinchoninic acid assay (Pierce BCA protein assay kit, Thermo Scientific). About 500 µg of protein was incubated with protein G beads which had been previously cross‐linked to either a SAFB1 antibody (AB1895, Invitrogen custom antibody services) or concentration‐matched rabbit IgG (Cell Signalling Technology). Lysates were rotated overnight at 4°C with antibody‐linked beads. Protein G beads bound to SAFB1 immunocomplexes were isolated from lysates using a magnetic rack and washed multiple times in lysis buffer. A sample of beads in solution from each experimental condition was reserved for analysis by Western blotting. Beads were pelleted and resuspended in BL/TG buffer from a ReliaPrep RNA Cell Miniprep kit (Promega) to dissociate immunocomplexes from protein G beads. RNA was purified from immunocomplexes using a ReliaPrep RNA Cell Miniprep Kit according to the manufacturer’s instructions.

### Quantification of ATXN1 RNA by qPCR

RNA was reverse transcribed using a GoScript Reverse Transcription System kit (Promega) using random primers according to the manufacturer’s instructions. cDNA was analyzed by qPCR with Power SYBR Green (Thermo Fisher Scientific) primers specific to ATXN1 (F:TCCAGCACCGTAGAGAGGAT, R:GCCCTGTCCAAACACAAAAA) with a StepOnePlusTM PCR system (Thermo Fisher Scientific). The relative level of ATXN1 expression was calculated by comparing CT values to that of a standard curve of serially diluted ATXN1 DNA and normalized per µg of SAFB1 protein. Relative ATXN1 expression for each condition was normalized to the mean ATXN1 expression level within each experimental replicate.

### Western blotting of protein lysates from RNA immunoprecipitation experiments

To confirm SAFB1 protein was successfully isolated using antibody‐linked protein G beads, samples of pre‐cleared input were run alongside samples of antibody‐linked and IgG‐linked protein G beads associated with immunocomplexes. Beads were resuspended in SDS sample buffer and heated at 95°C for 5 minutes to dissociate protein from the beads. Samples were run on an 8% polyacrylamide gel then transferred to a PVDF membrane using a Trans‐Blot Turbo Transfer System (Bio‐Rad). Membranes were blocked in 5% milk then probed with anti‐SAFB1 antibody (1:1000) (A300‐811 A, Bethyl Laboratories, UK) overnight at 4°C. Membranes were incubated with anti‐rabbit peroxidase‐conjugated secondary antibody (1:10 000) (GE Healthcare) and antibody signal visualized using ECL.

### Statistical analysis

Statistical tests were performed in GraphPad Prism (GraphPad Software Inc., version 7.04). For all tests, *P* < 0.05 represented statistical significance. Unpaired, two‐tailed *t*‐tests were used to compare data between the two groups. When comparing patient clinicopathological details between groups, the ages of control cerebellar tissue donors (cases 1‐4) were compared against the ages of SCA cerebellar tissue donors (cases 5‐9) using an unpaired, two‐tailed *t*‐test. The Post‐mortem delay (PMD) of the control and SCA cases was also compared by *t*‐test. *T*‐tests were also used to compare the age and PMD of control striatal tissue donors (cases 10‐16) and HD tissue donors (cases 17‐24). Results of *t*‐tests are reported as *t*(degrees of freedom) = *t*‐value, p value. All bar graphs represent the mean ± standard error of the mean (SEM). Where mean values are presented on graphs, the data from individual cases are represented by a circle (for control cases) or square (for disease cases) symbol. Relationships between two sets of data were analyzed using Pearson’s correlation tests.

## Results

### Abnormal expression of SAFB1 in SCA is a novel histopathological hallmark

**Figure 1:**
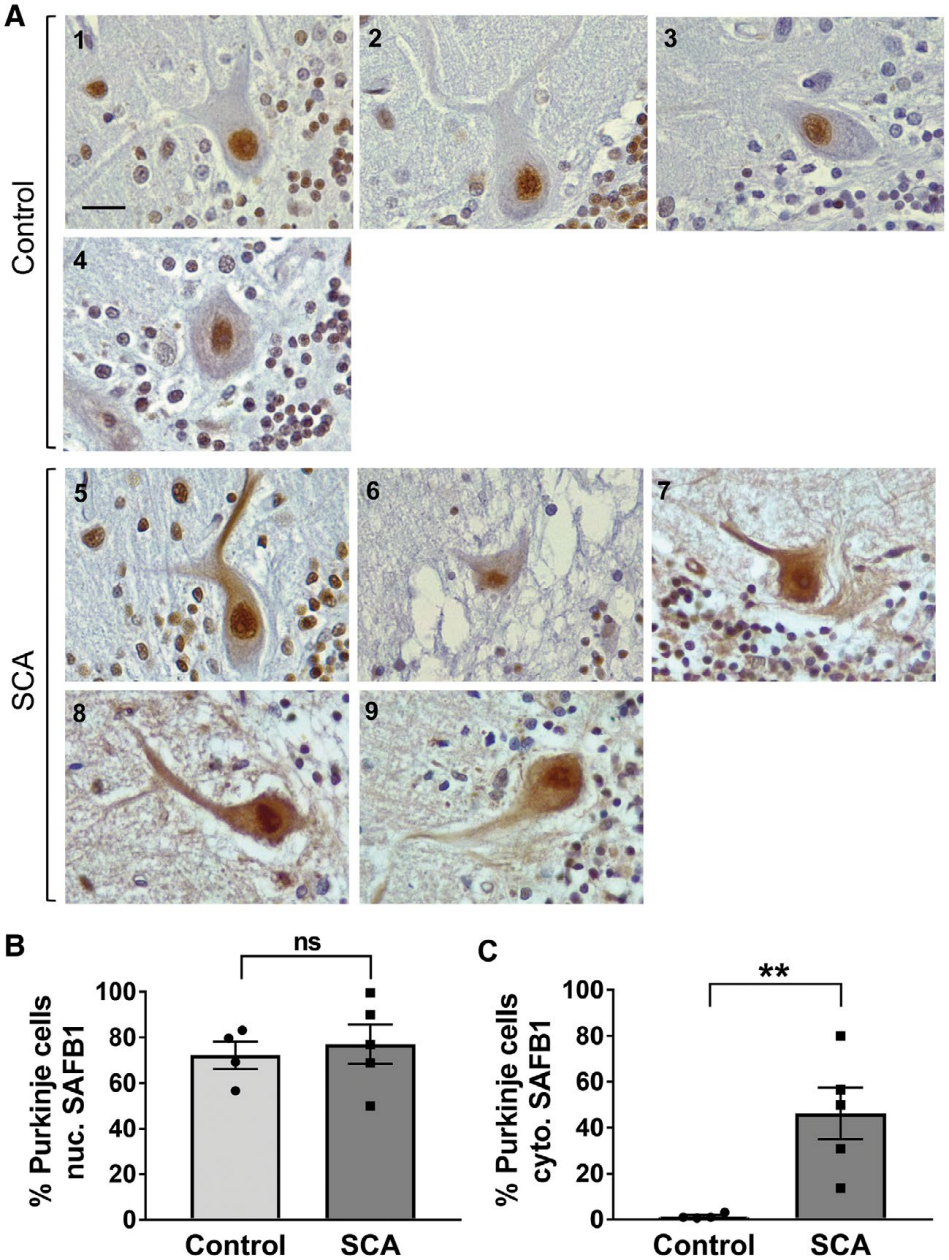
*Abnormal expressed of SAFB1 in cerebellar Purkinje cells is a feature of SCAs*. **A.** Representative images of Purkinje cells immunostained for SAFB1. In control cases, cells show SAFB1 expression in the nucleus but not in the cytoplasm. In SCA patients, Purkinje cells stain strongly for SAFB1 in the nucleus and feature abnormal SAFB1 expression in the soma and dendrites. Inset numbering represents the patient case number, scale bar is 25 µm. SAFB1 immunostaining was quantified by counting the percentage of Purkinje cells with immunopositivity in either the nucleus or cytoplasm. **B.** There is no difference between the percentage of cells positive for nuclear SAFB1 in SCA patients compared to control. **C.** There is a significant increase in the frequency of SAFB1 cytoplasmic immunopositivity in the SCA patient group compared to control (unpaired, two‐tailed *t*‐test *t*(7) = 3.496, *P* = 0.01). ns = not significant, **P < 0.01. Data are presented as mean ± SEM.

**Figure 2:**
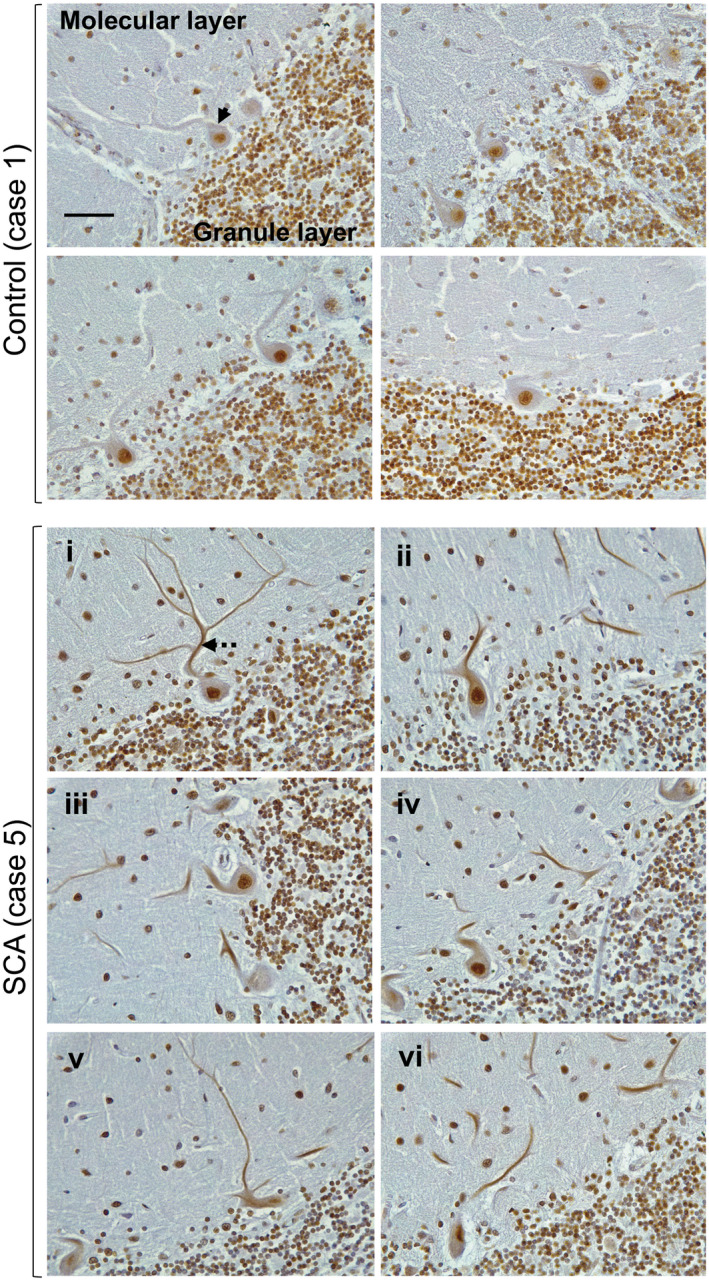
*Extensive abnormal SAFB1 expression in the cytoplasm of Purkinje cells in SCA1*. The photomicrographs shown are the representative images of cerebellar tissue from a Post‐mortem control case of a genetically diagnosed SCA1 patient immunostained for SAFB1. In control cerebellum, Purkinje cells in control tissue often feature SAFB1 immunostaining within the nucleus, but cytoplasmic SAFB1 staining is not present (arrow). In SCA1 cerebellum, Purkinje cells have cytoplasmic SAFB1 staining in the soma and dendrites (dotted arrow) in addition to strong nuclear staining. Cytoplasmic SAFB1 staining appears in SCA1 Purkinje cells with mild (**i–ii**), moderate (**iii–iv**) and substantial (**v–vi**) morphological alteration in comparison to control Purkinje cells. Scale bar is 50 µm.

Immunohistochemical characterization of SAFB1 expression was carried out in cerebellar tissue from patients diagnosed with SCA and compared to the cerebellum from control cases with no history of neurological disease (Tables 1 and S1). There was no significant difference in the age (*t*(7) = 0.27, *P* = 0.79) or Post‐mortem delay (*t*(6) = 1.57, *P* = 0.17) of the SCA patients when compared to the controls (unpaired, two‐tailed *t*‐tests). It should be noted that one of the patients was genetically confirmed as SCA1, one had a family history of autosomal dominant SCA and another was confirmed as having polyglutamine inclusions. Two of the patients were diagnosed as SCA patients based on presentation and Post‐mortem pathology (Table S1). The SAFB1 antibody used for immunostaining was previously shown to be specific for SAFB1 (Rivers *et al*, 2015) and control staining with no primary antibody was negative (Figure S1). SAFB1 immunopositivity in Purkinje cells was quantified by counting the percentage of Purkinje cells positive for SAFB1 across whole sections (Figure 1). Low power photomicrographs (Figure S2) show Purkinje cells within the PC layer juxtaposed with the molecular and granule cell layer (as indicated in Figure 2). The percentage of Purkinje cells with nuclear SAFB1 immunopositivity was not significantly different in the SCA patient tissue compared to control (Figure 1B). There was, however, a significantly higher percentage of Purkinje cells with cytoplasmic SAFB1 staining in SCA patients compared to controls (Figure 1C). We further characterized SAFB1 staining in a genetically diagnosed SCA1 patient (Table S1). SAFB1 immunoreactivity was observed within the nuclei of cells within the granular and molecular layers of the folia within both control and SCA1 cerebellum. In control tissue, SAFB1 staining in Purkinje cells was limited to within the nuclei and as in SCA patients, it was found to be expressed throughout the nucleus (Figures 1 and 2). In SCA1 Purkinje cells SAFB1 immunoreactivity was frequently present in both the nucleus and within the cytoplasm of the perikaryon and dendrites (Figure 2 and Table S2). Cytoplasmic SAFB1 staining was evident in SCA1 Purkinje cells with mild, moderate and substantial morphological alterations (Figure 2).

**Figure 3:**
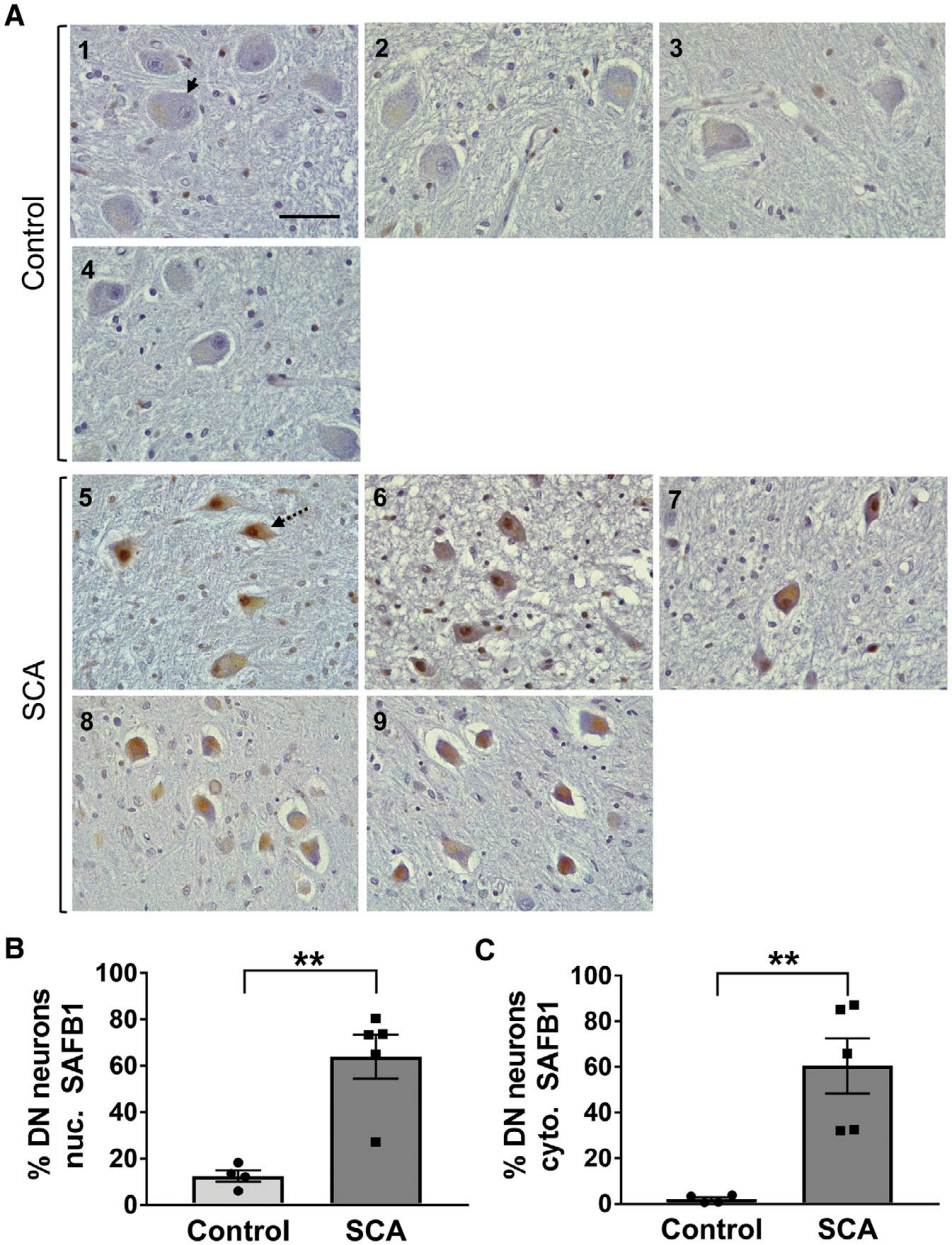
*SAFB1 expression is increased in the nucleus and cytoplasm of cerebellar dentate nucleus neurons in SCA patients*. **A.** Representative images of cerebellar dentate nucleus immunostained for SAFB1 show large principle neurons (identifiable by their morphology) within the neuropil of the dentate nucleus in control and SCA cases (arrow). Cells within SCA patient dentate nucleus appear smaller and shrunken in shape (dotted arrow) and show increased nuclear and cytoplasmic SAFB1 expression. Numbering represents patient case number, scale bar is 50 µm. **B‐C.** SAFB1 Immunoreactivity is expressed as the percentage of large principle neurons which are immuno‐positive for SAFB1 in either the nucleus or the cytoplasm. In SCA patients, a significantly higher percentage of neurons are positive for SAFB1 within the nuclei compared to control cases (unpaired, two‐tailed *t*‐test *t*(7) = 4.675, *P* = 0.0023) (**B**). Cytoplasmic SAFB1 is also expressed in a significantly higher percentage of neurons of SCA patients compared to control cases (unpaired, two‐tailed *t*‐test *t*(7) = 4.253, *P* = 0.0038) (**C**). **P < 0.01, ***P < 0.001. Data are presented as mean ± SEM.

In addition to atrophy and loss of Purkinje cells within the cerebellar cortex, the pathology of polyglutamine SCA can also affect the large principle neurons within the dentate nucleus of the cerebellar white matter. Notably, SAFB1 staining was not detected in the dentate neurons in the control cases (Figure 3A). However, there was a significantly increased expression of SAFB1 in both the nucleus (Figure 3A,B) and cytoplasm (Figure 3A,C) of dentate nuclei neurons in SCA patients (Table S2). The dentate nuclei neurons of SCA patients appeared smaller and shrunken and SAFB1 staining was diffused and distributed throughout the cell bodies (Figure 3A and Table S2) as in Purkinje cells. There is, however, less dentate neuron cell loss compared to that seen for Purkinje cells. Taken together, these data shows that abnormal cytoplasmic SAFB1 expression is a feature of two neuronal populations specifically affected by polyglutamine‐induced neurodegenerative damage in SCA.

**Figure 4:**
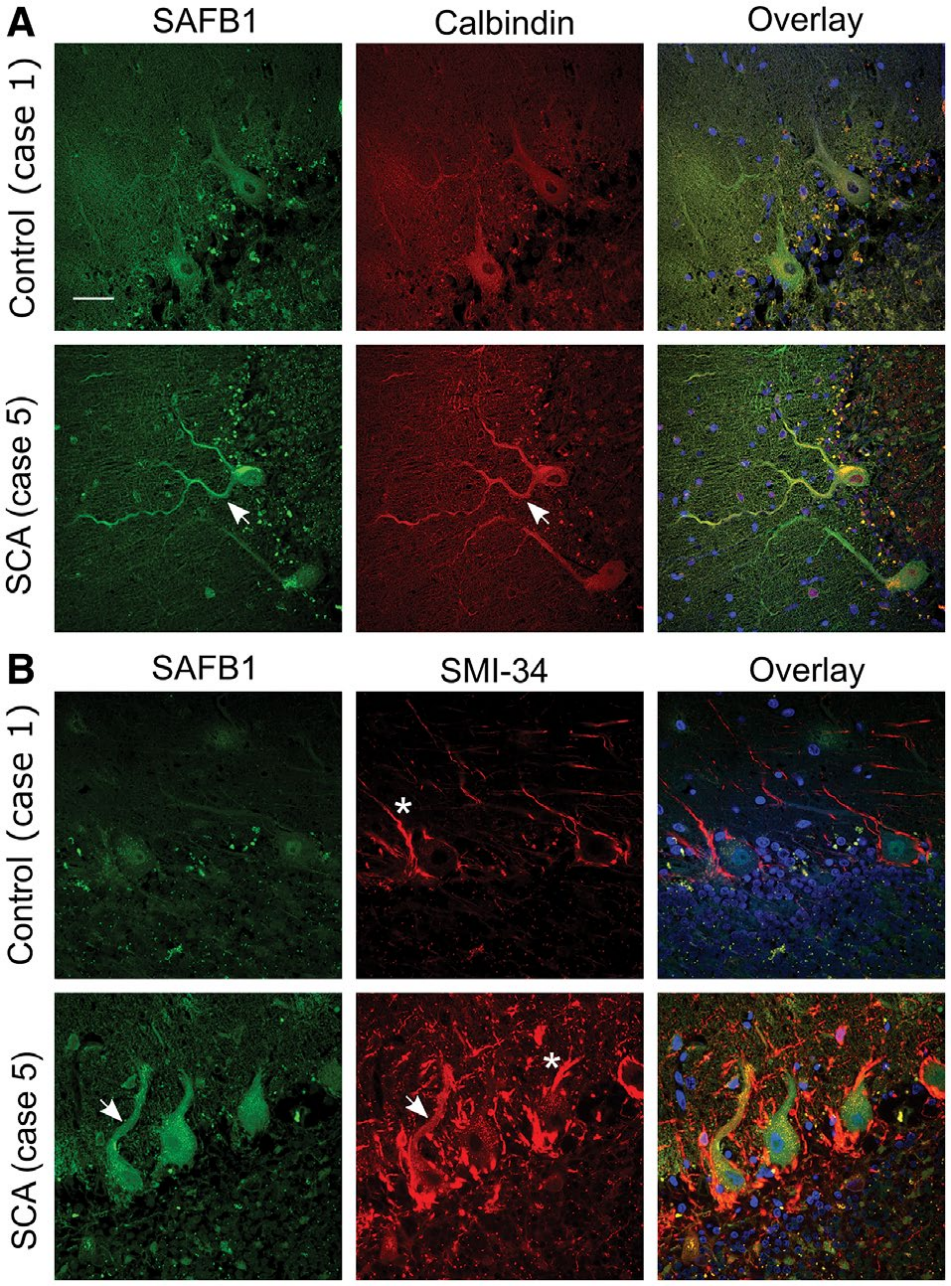
*SAFB1 colocalizes with calbindin and SMI‐34 in the cytoplasm of SCA Purkinje cells*. **A.** Calbindin immunopositivity specifies Purkinje cell soma and dendrites. SAFB1 staining is absent from the cytoplasm of control Purkinje cells but is colocalized with calbindin in SCA cases (arrows). Cell nuclei were stained using DAPI (blue). **B.** SMI‐34 staining, a marker of Purkinje cell damage colocalizes with SAFB1 in the soma and dendrites of SCA cerebellum (arrows) but not control tissue. SMI‐34 also stains basket cell projections (asterisk), which appear hypertrophied in SCA tissue compared to control. Scale bar is 50 µm.

To confirm that cytoplasmic SAFB1 staining was specific to Purkinje cells, dual immunofluorescent labeling was performed using antibodies against SAFB1 and calbindin‐D28k (a calcium‐binding protein which is widely used as a Purkinje cell marker). Representative images show that cytoplasmic SAFB1 was present in cerebellar Purkinje cells, as determined by co‐localization with calbindin immunoreactivity (Figure 4A). To assess whether cytoplasmic SAFB1 expression was localized to injured or damaged Purkinje cells in SCA, cerebellar sections were dual labeled for SAFB1 and SMI‐34. SMI‐34 is raised against hyperphosphorylated neurofilaments (Covance, USA) and been found to recognize intraneuronal pathological Tau paired helical filaments (PHF‐tau/Alzheimer disease tangles) (32) and to be a marker of Purkinje cell damage in Post‐mortem human brain (33). SCA Purkinje cells featuring hallmark morphological features of neurodegenerative injury (such as reduced perikaryon size and a deflated appearance to the nucleus) positive for SMI‐34 staining also contained abnormal cytoplasmic SAFB1 expression (Figure 4B). Immunofluorescent images show increased cytoplasmic SAFB1 staining relative to nuclear staining. This is likely a feature of the immunofluorescent staining, though it may also result from the low levels of SAFB1 staining seen in the nuclei of some control patients (Figure S3).

**Figure 5:**
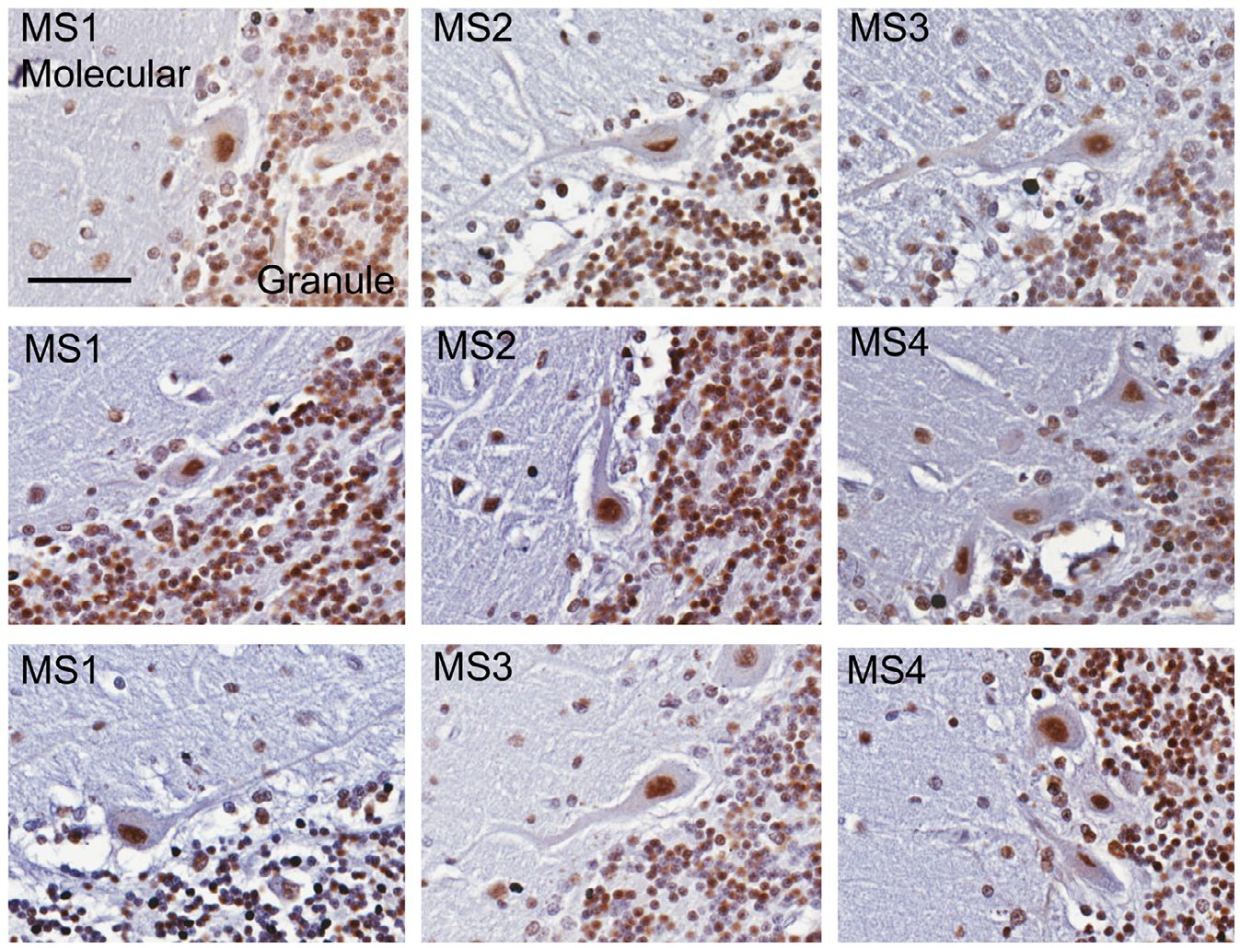
*SAFB1 expression is unaltered in the dystrophic Purkinje cells of MS patients*. Cerebellar Purkinje cells in patients with MS immunostained for SAFB1 (representative images from n = 4 patients, scale bar is 25 µm). SAFB1 staining within Purkinje cells with a typically dystrophic phenotype is nuclear without immunoreactivity in the cytoplasm of the cell soma or dendrites. The percentage of Purkinje cells positive for SAFB1 staining in the nucleus in each patient group is as follows (mean ± SEM); Control (72.2 ± 5.6), MS (83.3 ± 10.7), SCA (77.1 ± 8.6). The percentage of Purkinje cells positive for SAFB1 in the cytoplasm per group is as follows; Control (1.4 ± 0.6), MS (5.1 ± 1.9) SCA (46.2 ± 11.3).

Previous studies have shown that Purkinje cell degeneration and loss occurs in patients with multiple sclerosis (MS) (33, 45). Therefore, to assess whether abnormal cytoplasmic SAFB1 expression is specifically associated with Purkinje cell injury induced by polyglutamine disease, cerebellar tissue sections from four patients diagnosed with multiple sclerosis were immunolabeled for SAFB1. Multiple sclerosis causes Purkinje cell damage and degeneration, but this is not caused by polyglutamine pathology. Analysis of Purkinje cells across whole cerebellar sections including those with a dystrophic phenotype showed that SAFB1 immunostaining was confined to the nucleus, following the same pattern to that of control patients (Figure 5). We also immunostained substantia nigra sections with SAFB1 and found there was no difference in expression between controls and Parkinson’s patients (Figure S4).

**Figure 6:**
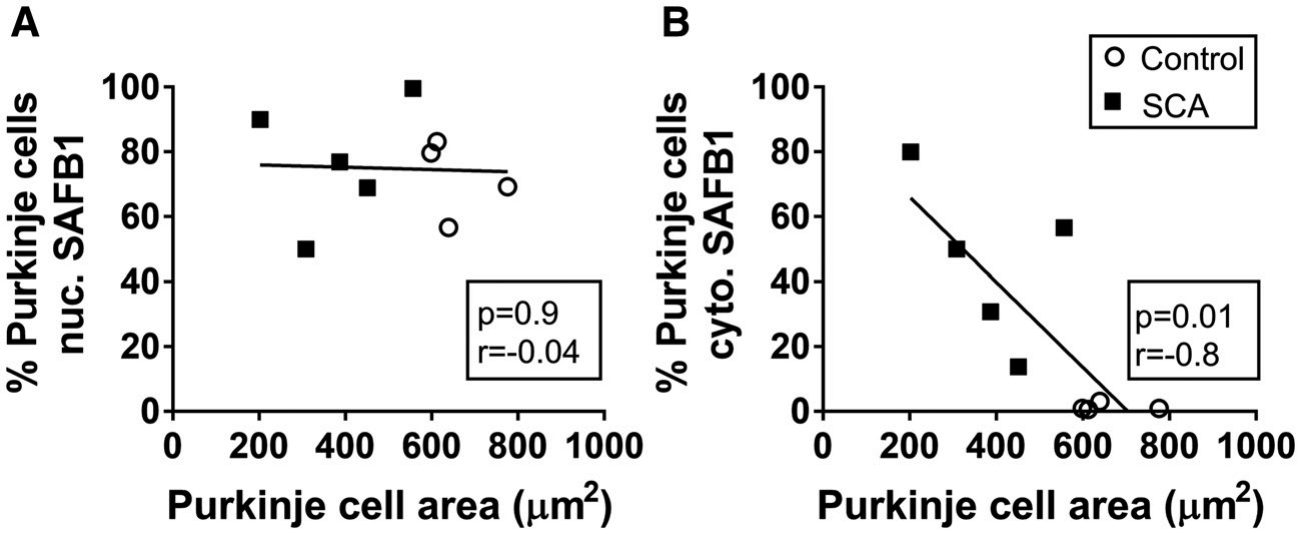
*SAFB1 expression correlates with decreased Purkinje cell area in SCA patients*. There was no significant correlation between SAFB1 staining in the nucleus and Purkinje cell area (**A**). However, increased cytoplasmic SAFB1 staining was correlated significantly with a decrease in Purkinje cell perikaryon area (**B**). Statistical analyses were carried out using Pearson correlation tests and the P and r values are depicted within each graph.

The neuropathology of autosomal dominant spinocerebellar ataxias caused by polyglutamine expansion repeats has been well characterized and frequent pathological hallmarks include a reduction in the number of Purkinje cells (Table S1) and reduction in Purkinje cell size. To determine whether there was a relationship between Purkinje cell histopathological characteristics and the frequency of SAFB1 staining, Pearson correlative analysis was carried out (Figure 6). There was no significant correlation between Purkinje cell perikaryon area and nuclear SAFB1 staining (Figure 6A). However, there was a significant correlation between SAFB1 staining in the cytoplasm and Purkinje cell perikaryon area (Figure 6B). Taken together the data show that increasing frequency of abnormal SAFB1 in the cytoplasm is associated with Purkinje cell injury, as measured by shrinkage of the cell soma and loss of cell number. In a single SCA case, who died as a result of cancer, there was a larger number of surviving Purkinje cells (compared to 4 other SCA patients, Table S1) and over 56% of these cells stained positive for cytoplasmic SAFB1. The presentation of SAFB1 pathology in this single SCA case suggests that changes in SAFB1 expression occur relatively early in the disease process.

**Figure 7:**
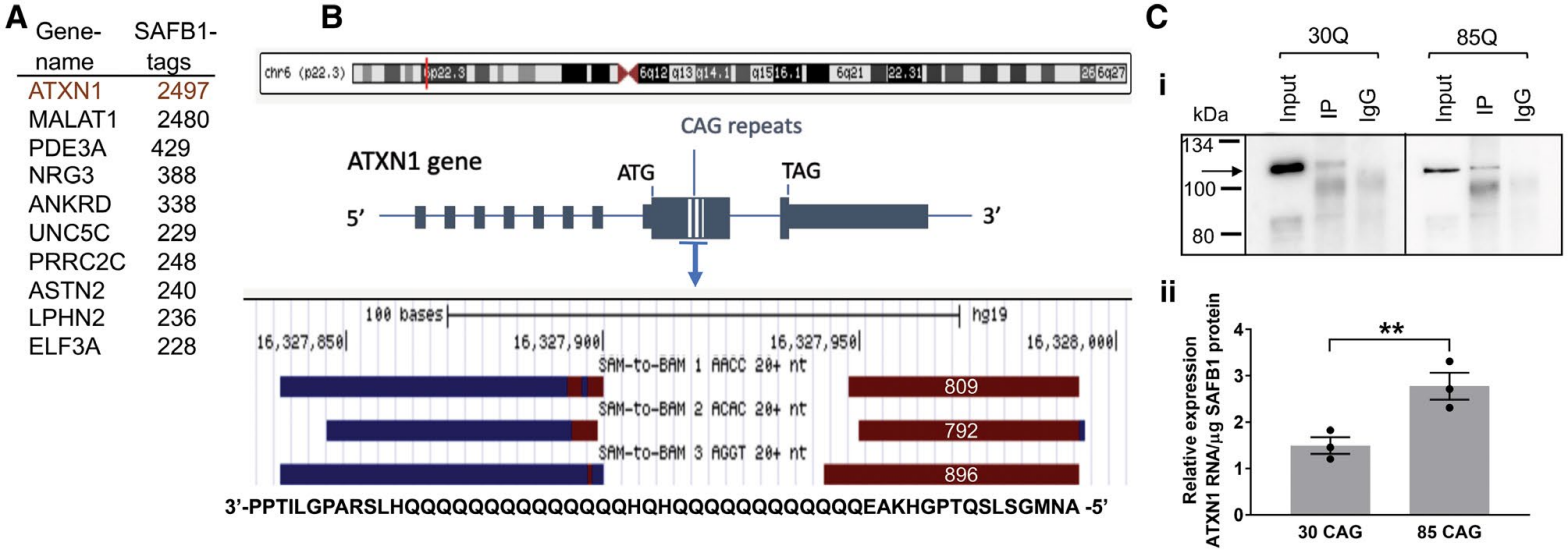
*Increased binding of SAFB1 to Ataxin 1 with an expanded glutamine tract*. **A.** The top 10 most SAFB1 bound RNA transcripts with the total number of iCLIP SAFB1 cross‐link sites from three independent experiments. **B.** The red line depicts the location of the *ATXN1* gene on chromosome 6. The majority of the SAFB1 binding sites occur in RNA encoded by exon 8 of *ATXN1* and in the region containing CAG repeat sequence (blue hatched lines). A screengrab taken from the genome browser shows the sense (brown) and antisense (blue) SAFB1 iCLIP binding sites. The binding sites relative to the amino‐acids encoded by the SAFB1 bound sequences are shown in a 3′‐5′ orientation. Note the SAFB1 cross‐link site will correspond to the amino‐acid most 5′ to the sense sequence and 3′ to the antisense sequence. The numbers within the schematic of the sense binding sites show the number of SAFB1 cross‐link sites in each of these individual experiments. **C** **i.** Western blotting of protein lysates from RNA immunoprecipitation experiments, probed for SAFB1. SAFB1 is present in both ATXN1‐30Q transfected and ATXN1‐85Q transfected cell lysates immunoprecipitated using a SAFB1 antibody (IP). There is no SAFB1 protein detected in IgG control lysates (IgG). Expected molecular weight for SAFB1 is 102KDa (arrow). **ii.** Relative levels of ATXN1 RNA bound to SAFB1 protein. SAFB1 protein was immunoprecipitated from HeLa cells transfected with plasmids containing ATXN1‐30CAG (physiological) or ATXN1‐85CAG (pathological expansion). The relative expression level of ATXN1 RNA associated with SAFB1 protein is significantly increased in ATXN1‐85 CAG compared to ATXN1‐30 CAG (unpaired, two‐tailed *t*‐test *t*(4) = 3.76, *P* = 0.01). Data are presented as mean ± SEM.

We used individual‐nucleotide resolution crosslinking and immunoprecipitation (iCLIP) to identify SAFB1 RNA binding partners and found that ATXN1 RNA contained the greatest number of SAFB1 RNA binding sites (Figure 7A). The SAFB1 iCLIP tags all mapped to ATXN1 RNA encoded by exon 8 and the crosslink sites indicated SAFB1 bound ATXN1 primarily at the start of the CAG repeat region (Figure 7B), though sites were also found within the CAG repeats. In addition, our results showed that SAFB1 bound antisense ATXN1 (ATXN‐AS1) RNA within the GTC repeat region (31). To investigate whether increasing the number of CAG repeats would alter SAFB1 binding, we transfected neuroblastoma cells with constructs encoding full‐length ATXN1 mRNA constructs with either 30 (control ATXN1‐30Q) or 85 polyglutamine (disease ATXN1‐85Q) repeats (ref) and carried out SAFB1 immunoprecipitation followed by qPCR for ATXN1 (Figure 7C). Our results showed that SAFB1 bound non‐pathogenic ATXN1‐30Q mRNA, however, this association was significantly increased when there was a pathological polyQ expansion (ATXN85Q). To control for binding mediated by endogenously expressed ATXN1 in HeLa cells, control cells not transfected with an ATXN1 plasmid were included in each experiment. In these experiments, qPCR analysis showed that the relative quantity of endogenous ATXN1 RNA bound to SAFB1 protein in non‐transfected cells was extremely low (compared to that in cells expressing exogenous ATXN1) and did not complicate interpretation of the assay. In addition to this data, we have conducted a TMT proteomic analysis of proteins pull‐down with a SAFB1 specific antibody and we did not find SAFB1 bound ATXN1 protein (*unpublished observations)*. Furthermore, when EGFP tagged ATXN1 constructs with 2, 30 and 52Q repeats are expressed in neuroblastoma cells they do not bind SAFB1 *(unpublished observations)*.

### SAFB1 expression is abnormal in striatal Huntington’s disease neurons

**Figure 8:**
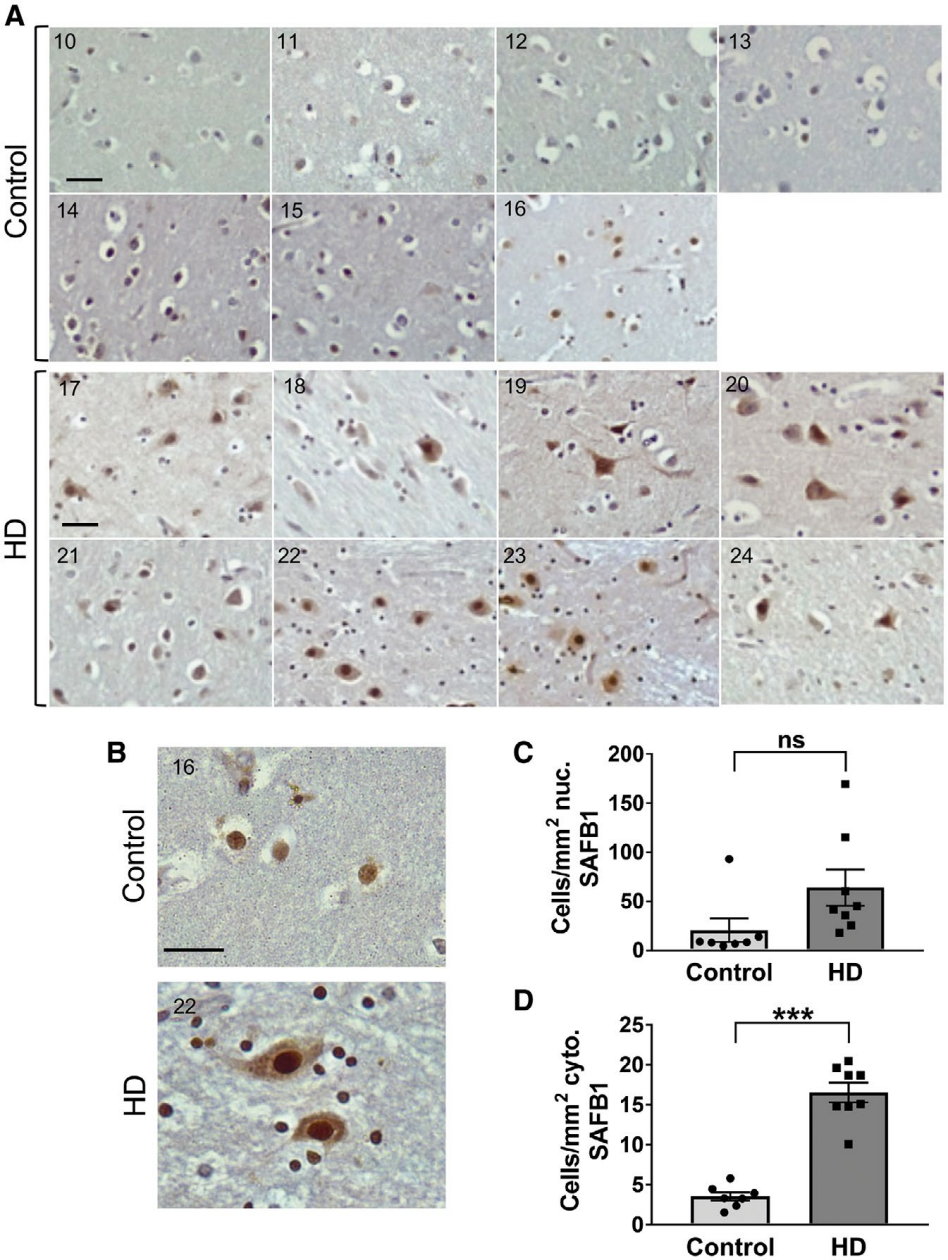
*SAFB1 expression is altered in the striatal neurons of HD patients*. **A.** Representative images from individual cases showing the distribution of SAFB1 staining within striatal cells in control and HD patients. **B.** A representative higher magnification image showing SAFB1 staining in control tissue is mostly confined to the nucleus of larger cells within the striatum. SAFB1 staining in HD striatum is stronger and present in both the nuclei and cytoplasm of larger cells (MSN). Numbers in individual photomicrographs represent the patient case number. **C.** There is no significant difference in the number of cells with nuclear SAFB1 staining in HD cases compared to controls (unpaired, two‐tailed *t*‐test *t*(13) = 1.9, *P* = 0.08). **D.** There is a significantly higher number of cells positive for cytoplasmic SAFB1 in the striatum of HD patients compared to in control cases (unpaired, two‐tailed *t*‐test *t*(13) = 9.266, *P* < 0.0001). Data are presented as mean ± SEM. Scale bars are 25 µm.

**Figure 9:**
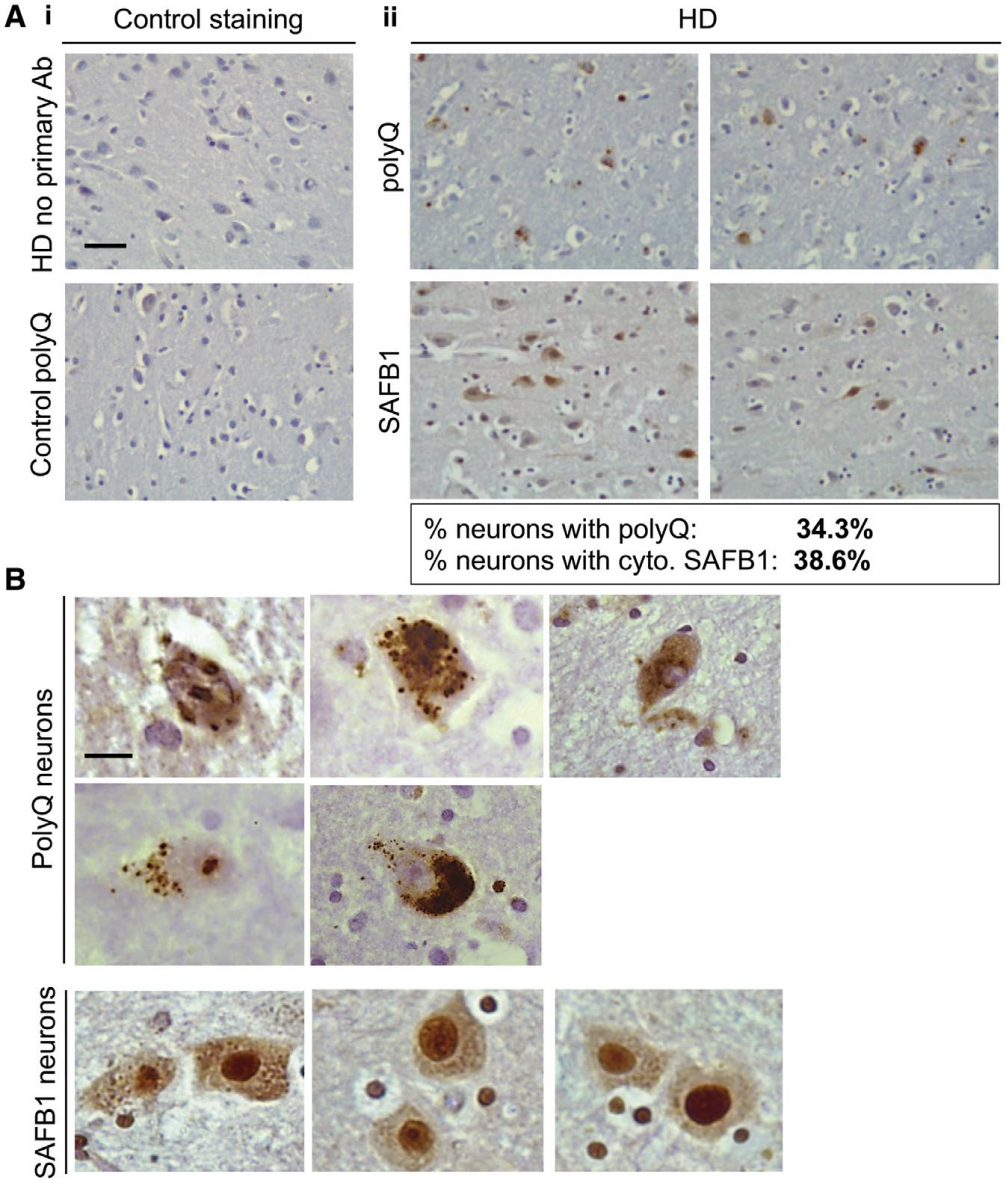
*Frequency and localization of SAFB1 staining compared with polyglutamine inclusions in HD*. **A** **i.** No primary antibody control staining of striatal tissue from an HD patient (upper panel). Polyglutamine immunostaining using antibody 1C2 in the striatum of a control patient shows no immunoreactivity (lower panel). **ii.** Representative low‐magnification images of polyglutamine and SAFB1 staining in separate, serial striatal sections of an HD patient (case 23). The percentage of neurons positive for cytoplasmic SAFB1 and for polyQ is indicated below the images. **B.** High magnification images show that polyQ aggregates/inclusions occur in both the nucleus and cytoplasm. SAFB1 staining is generally homogenous, with strong staining throughout the nucleus and an even distribution of staining with small puncta present throughout the cytoplasm. Scale bar in (**A**) is 50 µm, in (**B**) is 10 µm.

As SAFB1 protein expression has not previously been characterized in any human polyglutamine disorders, following our findings in SCA, we next investigated SAFB1 expression in HD, the most common neurodegenerative disease caused by CAG expansion repeats. Post‐mortem striatal tissue was obtained from a cohort of patients diagnosed with HD (ranging between Vonsattel grade 2‐4) and from control cases with no history of neurological disease (Figure 8A,B, Table 1). There was no significant difference in age (*t*(13) = 0.69, *P* = 0.5) or Post‐mortem delay (*t*(13) = 0.92, *P* = 0.38) when HD patients were compared to controls (unpaired, two‐tailed *t*‐tests). SAFB1 immunostaining was evident in the nuclei of striatal cells and there was no significant difference in the number of cells positive for nuclear SAFB1 in HD patient tissue compared to control (Figure 8C). However, in Huntington’s cases, strong cytoplasmic SAFB1 staining was present in the cells of the striatum and this was not found in controls (Figure 8D). In addition, the SAFB1 staining in the cytoplasm was found to occur mainly in large dystrophic cells (presumably medium spiny neurons) and this was invariably accompanied by increased SAFB1 nuclear staining. We next compared the frequency and localization of SAFB1 staining with that of polyglutamine inclusions. Analysis of low‐magnification images found that the frequency of abnormal neuronal SAFB1 staining and polyglutamine inclusions was similar (38.6% and 34.3%, respectively; Figure 9A). High‐magnification images (Figure 9B) showed that polyglutamine staining was present in both the nucleus and cytoplasm of degenerating neurons and that it generally presented as concentrated large puncta/aggregates. In contrast, SAFB1 staining was more homogenous, with strong staining throughout the nucleus and an even distribution of staining with small puncta present throughout the cytoplasm. These differing staining patterns strongly suggest that the SAFB1 protein is not closely associated with polyglutamine aggregates in HD neurons.

## Discussion

In this study, we investigated the expression of the DNA and RNA binding protein SAFB1 in the Post‐mortem human brains of SCA, HD, PD and MS patients. We found that SAFB1 expression was increased in the nucleus and mislocalized to the cytoplasm in the Purkinje and cerebellar dentate cells of SCA patients and in the striatal neurons of HD patients. We further showed that abnormal SAFB1 cytoplasmic expression co‐localized with a Purkinje cell‐specific marker and col‐localized with SMI‐34 an antibody that labels intraneuronal pathological PHF‐tau (32) and has correlated with Purkinje cell damage (15). Cerebellar dentate cell degeneration and loss also occurs in SCA patients (16) and dentate neurons displaying a shrunken phenotype correlated with increased SAFB1 expression in both the nucleus and cytoplasm. The change in nuclear and cytoplasmic SAFB1 expression under these disease conditions was dramatic as there was no SAFB1 staining in the dentate and striatal neurons of control patients. Furthermore, studies have shown that Purkinje cell loss and the mislocalization of RBPs to the cytoplasm of neurons occurs in patients with MS (33, 38, 45). However, we found that there was no abnormal SAFB1 expression in the dystrophic Purkinje cells of MS patients or in the substantia nigra of PD patients indicating that abnormal SAFB1 may be specific to diseases characterized by glutamine expansions. Interestingly, in Huntington’s cases, the abnormal SAFB1 nuclear and cytoplasmic staining pattern differed to that of polyglutamine inclusions suggesting the two proteins do not interact. However, we did show that SAFB1 binds ATXN1 RNA adjacent to and within the glutamine repeat region and that there was significantly increased SAFB1 binding to Ataxin 1 RNA containing an expanded pathogenic polyglutamine tract. This suggests that SAFB1 may bind RNA motifs adjacent to polyglutamine tracts and within polyglutamine tracts, for example, due to the formation of a G‐quadruplex structure. RBPs often interact with other RBPs and this allows distinct RNA motifs in close proximity to be bound simultaneously and increases interaction specificity. Hence, the formation of long glutamine tracts may result in an RNA structure that creates multiple SAFB1 (and other RBP) binding sites. We found SAFB1 expression was abnormal in SCA and HD patients but was unaltered in MS or PD patients. Though it should be noted that while all the SCA patients used had extensive cerebellar/Purkinje cell pathology only three of the five were confirmed by genetic testing/IC2 immunoreactivity as being polyglutamine expansion/inclusion positive. It is, therefore, important that further studies are conducted to investigate SAFB1 pathology in SCA patients with confirmed polyglutamine phenotypes. In this regard, it would also be informative to investigate the association of SAFB1 pathology with markers that characterize other non‐polyglutamine expansion neurodegenerative conditions. Overall, as SAFB1 was strongly associated with HD and SCA disease phenotypes and considering the important roles it plays in regulating neuronal gene expression these data strongly suggest SAFB1 contributes to the pathology of polyglutamine illnesses.

SAFB1 is a multifunctional DNA and RNA binding protein implicated in the control of gene expression (24, 35, 40), DNA repair (2) apoptosis (20) and the formation of nuclear stress bodies (7). SAFB1 is known to interact with Sam 68, hnRNP K, SRSF1, FUS and Matrin 3 (28, 36, 46) and these interaction partners have been implicated in the pathology of repeat disorders such as Fragile X‐associated Tremor/Ataxia Syndrome (FXTAS) (39), SCA10 (44) and ALS/FTD (10, 42, 46). Considering the functions of SAFB1, it is evident that its upregulation and/or ectopic cytoplasmic expression in neurons of patients with polyglutamine expansion disorders would contribute to disease pathology. First, the altered expression of SAFB1 in the nucleus is likely to result in major changes in gene transcription, splicing and RNA transport and these will have major functional consequences. For instance, we have shown increased expression of SAFB1 alters dendritic function (35). SAFB1 organizes chromatin structure via phase separation (12) and the altered SAFB1 expression seen in polyglutamine disorders may, therefore, result in changes in heterochromatin stability. Mutations in FUS and matrin‐3 are associated with ALS and SAFB1 have been shown to regulate the interaction of FUS with chromatin and bind directly with Matrin 3 (also a nuclear matrix‐associated protein). Expression of the normally nuclear SAFB1 RNA binding protein in the cytoplasm will sequester coding and non‐coding RNAs and RNA binding proteins and lead to compromised neuronal function. In addition, we have shown that the SAFB1 paralogue, SAFB2 is expressed in axons and dendritic spines (28) and hence SAFB1 is likely to interfere with SAFB2 mediated RNA transport. Recently, the altered expression of DNA repair genes was implicated in polyglutamine tract expansion and the modification of age of neurological onset in HD and SCAs (11, 18). SAFB1 renders chromatin permissive to DNA repair and depending on the availability of cofactors its upregulation could promote or inhibit DNA repair. Investigating whether the up or down‐regulation of DNA repair modifiers such as SAFB1 accelerated or delayed the age of onset in models of HD/SCAs could, therefore, identify new therapeutic approaches.

The mechanism by which SAFB1 expression is altered in SCA and HD is uncertain. The increased cytoplasmic and nuclear SAFB1 expression seen (e.g. in cerebellar neurons and dentate neurons, respectively) may be due to: (i) it binding to transcribed and/or translated polyglutamine repeats; (ii) increased transcription mediated by feedback control; (iii) association with one or more protein partners altered in polyglutamine disorders. We found that there is significantly increased binding of SAFB1 to ATXN1 RNA encoding an expanded glutamine tract. This suggests that the mislocalization (and possibly up‐regulation) of SAFB1 may be linked to the altered processing and transport of RNA containing pathological polyglutamine tracts. This hypothesis is supported by the observation that there is altered nucleocytoplasmic transport of RBPs in polyglutamine expansion diseases and ALS (3, 8). SRSF1 (a known protein‐binding partner of SAFB1) mediates the export of repeat‐RNA to the cytoplasm (10). Furthermore, the expression of ALS‐linked FUS mutants has previously been reported to mediate the movement of SAFB1, Matrin‐3 and FUS to the cytoplasm (46). This altered SAFB1 expression could result from an inhibition of nuclear import or increased co‐export with a binding partner. Further studies to identify by what mechanism SAFB1 expression is altered and to what degree it contributes to early disease pathogenesis may highlight new therapeutic approaches for polyglutamine diseases.

## Conclusions

Our novel results show that the important gene regulatory protein SAFB1 is abnormally expressed in neurons from patients with polyglutamine expansion disorders and its altered expression is likely to cause neuronal pathology due to a loss of nuclear function and/or gain of function in the cytoplasm. Considering SAFB1 interacts with proteins (e.g. Sam 68, Matrin 3, hnRNP K & SRSF1) implicated in the etiology of human neurodegenerative conditions, these findings support the hypothesis that altering the interconnected networks of RNA binding proteins strongly contributes to the etiology of human neurodegenerative conditions.

## Ethics approval and consent to participate

Post‐mortem sections from patients diagnosed with spinocerebellar ataxia (SCA), Huntington’s disease (HD) and control cases with no history of neurological disease were obtained from the MRC London Neurodegenerative Diseases Brain Bank (ethical approval LNDBB 1778). Tissue was also obtained from the Multiple Sclerosis Tissue Bank, Imperial College, London, UK (ethical approval 08/MRE09/31).

## Consent for publication

Not applicable.

## Conflict of interest

The authors declare that they have no competing interests.

## Author contributions

NB and JU developed the concept of the study; NB, JU, KK, NA and AW designed experiments; NB, KK and HS performed experiments; NB, KK collected and analyzed data; NB, KK, NA and AW wrote the manuscript. All authors read and approved the final manuscript.

## Supporting information


**Figure S1.** SAFB antibody controls. Lysate from HeLa cells transfected with siRNAs against SAFB1 or SAFB2 was run by SDS‐PAGE and probed using antibodies against SAFB1 or SAFB2 (**A**). Both antibodies specifically recognise a reduction in SAFB1 expression. No primary antibody control striatal and cerebellar tissue sections have no DAB reactivity, indicating that there is no non‐specific detection from either the ABC detection system or from the sections themselves (**B**). Scale bars are 50 μm.

**Figure S2.** SAFB1 staining in the cerebellum of control and SCA patients (higher magnification images are shown in Figure 1). These representative images show Purkinje cell staining in relation to the cerebellar molecular and granule cell layers. Inset numbering represents the patient case number. Scale bar = 50 μm.

**Figure S3.** SAFB1 staining of cerebellar control patient sections. (**A**) A control patient Purkinje cell positive for SAFB1 immunofluorescent nuclear staining. Arrows indicates Purkinje cell nucleus. GL = granule cell layer. (**B**) Control patient Purkinje cells negative for SAFB1 expression in the nucleus (immunohistochemical DAB staining). Arrow indicates Purkinje cell nucleus. GL = granule cell layer.

**Figure S4.** Formalin fixed paraffin embedded sections from control and Parkinson’s disease (PD) substantia nigra, immunostained for SAFB1. Overall levels of SAFB1 staining in the substantia nigra are low compared to the cerebellum and striatum. There is no clear difference in nuclear SAFB1 staining in control patients compared to PD patients. There is no evidence of cytoplasmic SAFB1 staining in control or PD patient substantia nigra. 40× scale bar is 50 μM, magnified region scale bar is 25 μm.

**Table S1.** Spinocerebellar ataxia (SCA) patient diagnosis and pathology data.

**Table S2.** Purkinje and dentate neurons counted and number of cells positive for nuclear and/or cytoplasmic SAFB1 staining.

## Data Availability

All data generated or analyzed during this study are included in this published article [and its supporting information files].
