# Supplementary figures and images for: Abnormal scaffold attachment factor 1 expression and localization in spinocerebellar ataxias and Huntington’s chorea
Source: Brain Pathol. 2020 Jul 13;30(6):1041–55. doi: 10.1111/bpa.12872 (PMC8018166; doi:10.1111/bpa.12872)

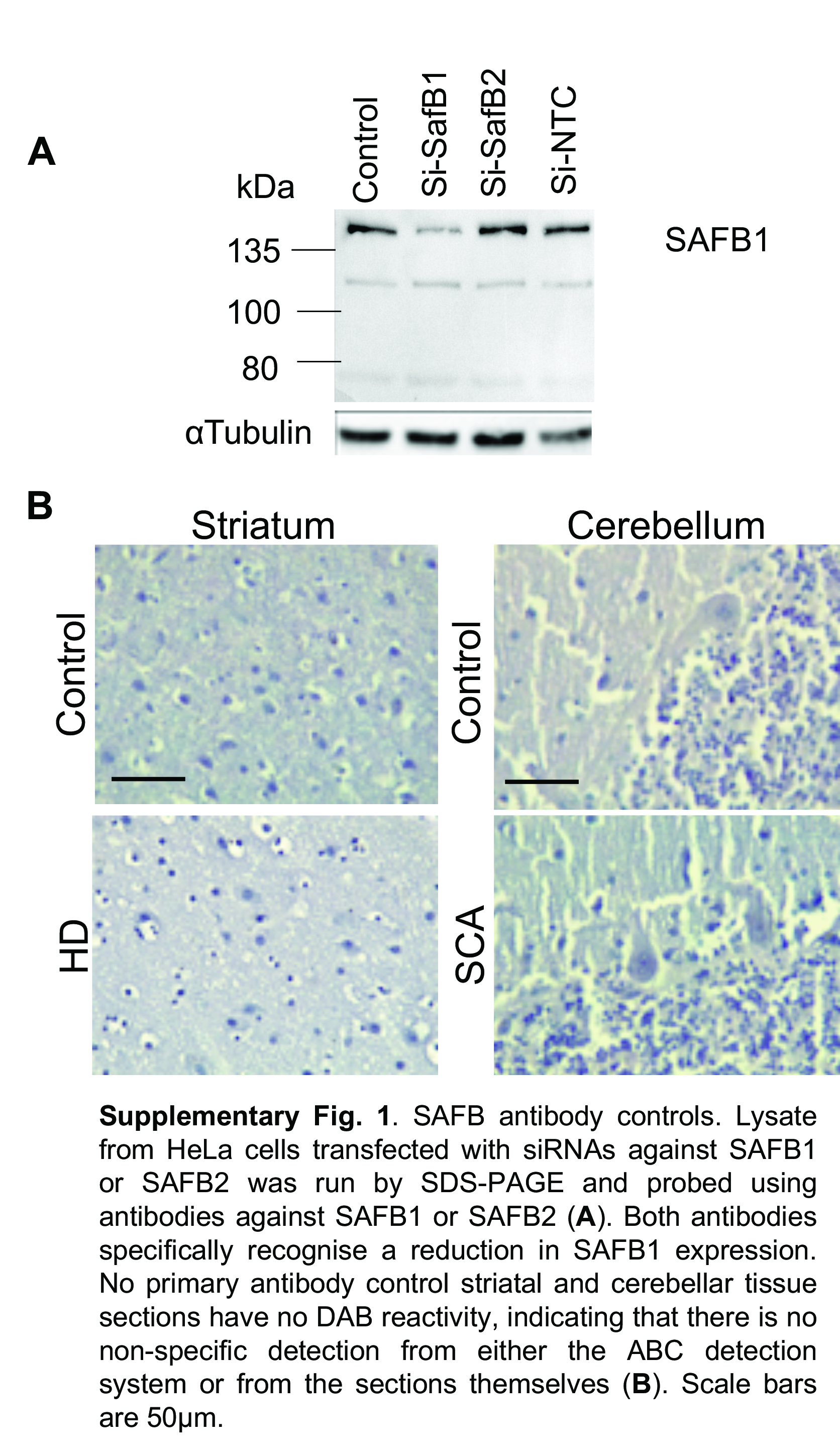

Supplement: Supplementary file 1 — Figure S1. SAFB antibody controls. Lysate from HeLa cells transfected with siRNAs against SAFB1 or SAFB2 was run by SDS‐PAGE and probed using antibodies against SAFB1 or SAFB2 (A). Both antibodies specifically recognise a reduction in SAFB1 expression. No primary antibody control striatal and cerebellar tissue sections have no DAB reactivity, indicating that there is no non‐specific detection from either the ABC detection system or from the sections themselves (B). Scale bars are 50 μm. [file BPA-30-1041-s006.tif]

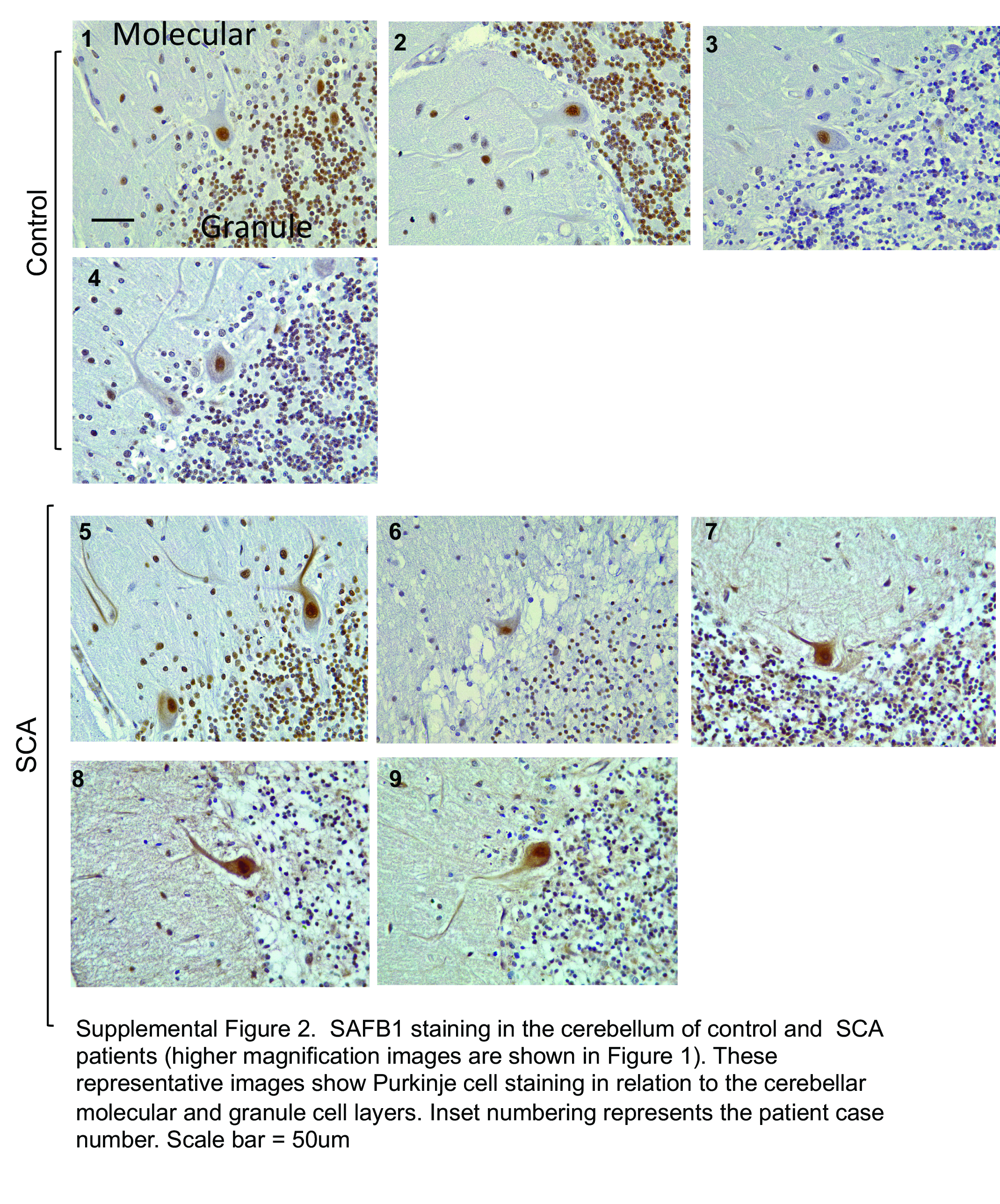

Supplement: Supplementary file 2 — Figure S2. SAFB1 staining in the cerebellum of control and SCA patients (higher magnification images are shown in Figure 1). These representative images show Purkinje cell staining in relation to the cerebellar molecular and granule cell layers. Inset numbering represents the patient case number. Scale bar = 50 μm. [file BPA-30-1041-s005.tif]

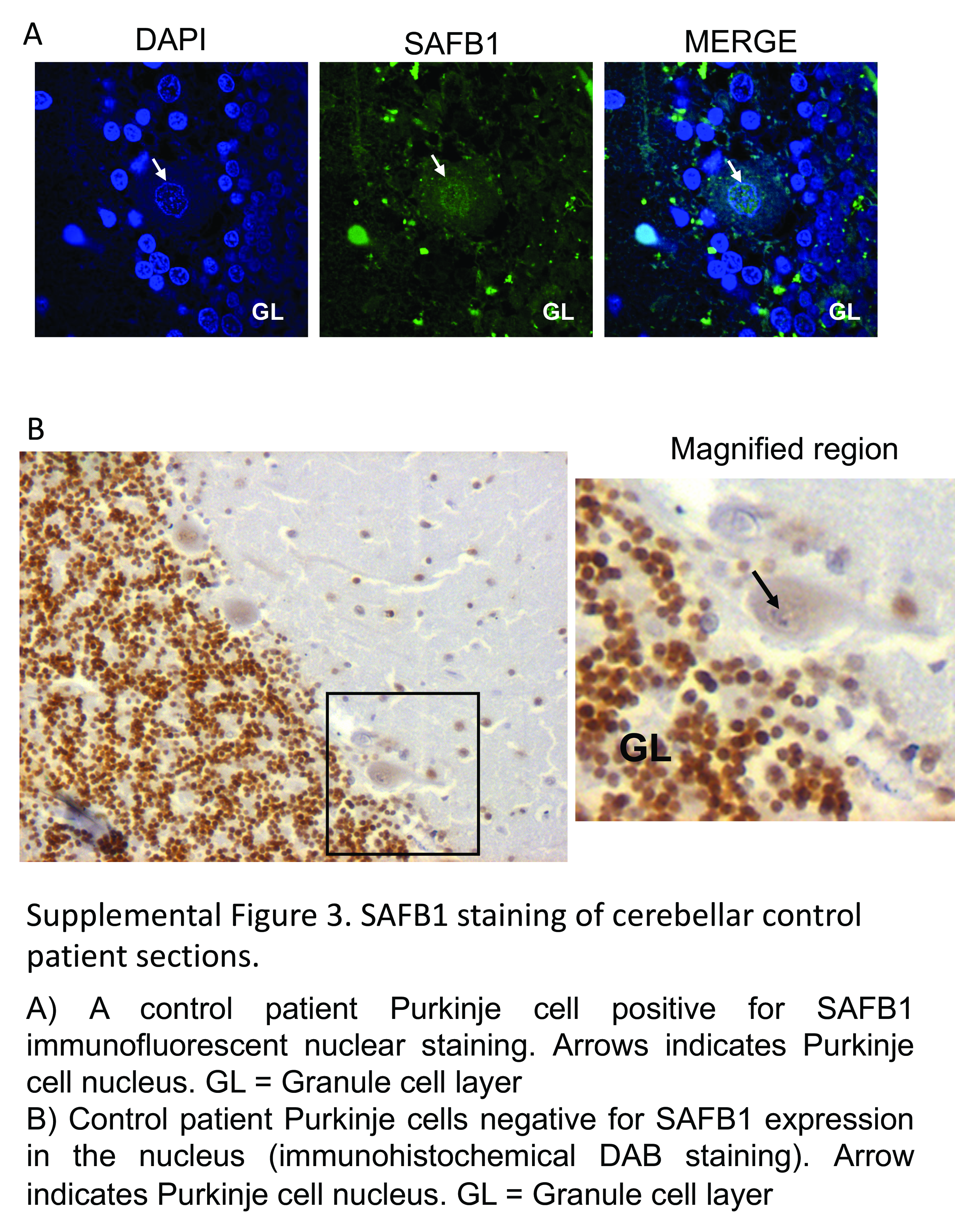

Supplement: Supplementary file 3 — Figure S3. SAFB1 staining of cerebellar control patient sections. (A) A control patient Purkinje cell positive for SAFB1 immunofluorescent nuclear staining. Arrows indicates Purkinje cell nucleus. GL = granule cell layer. (B) Control patient Purkinje cells negative for SAFB1 expression in the nucleus (immunohistochemical DAB staining). Arrow indicates Purkinje cell nucleus. GL = granule cell layer. [file BPA-30-1041-s004.tif]

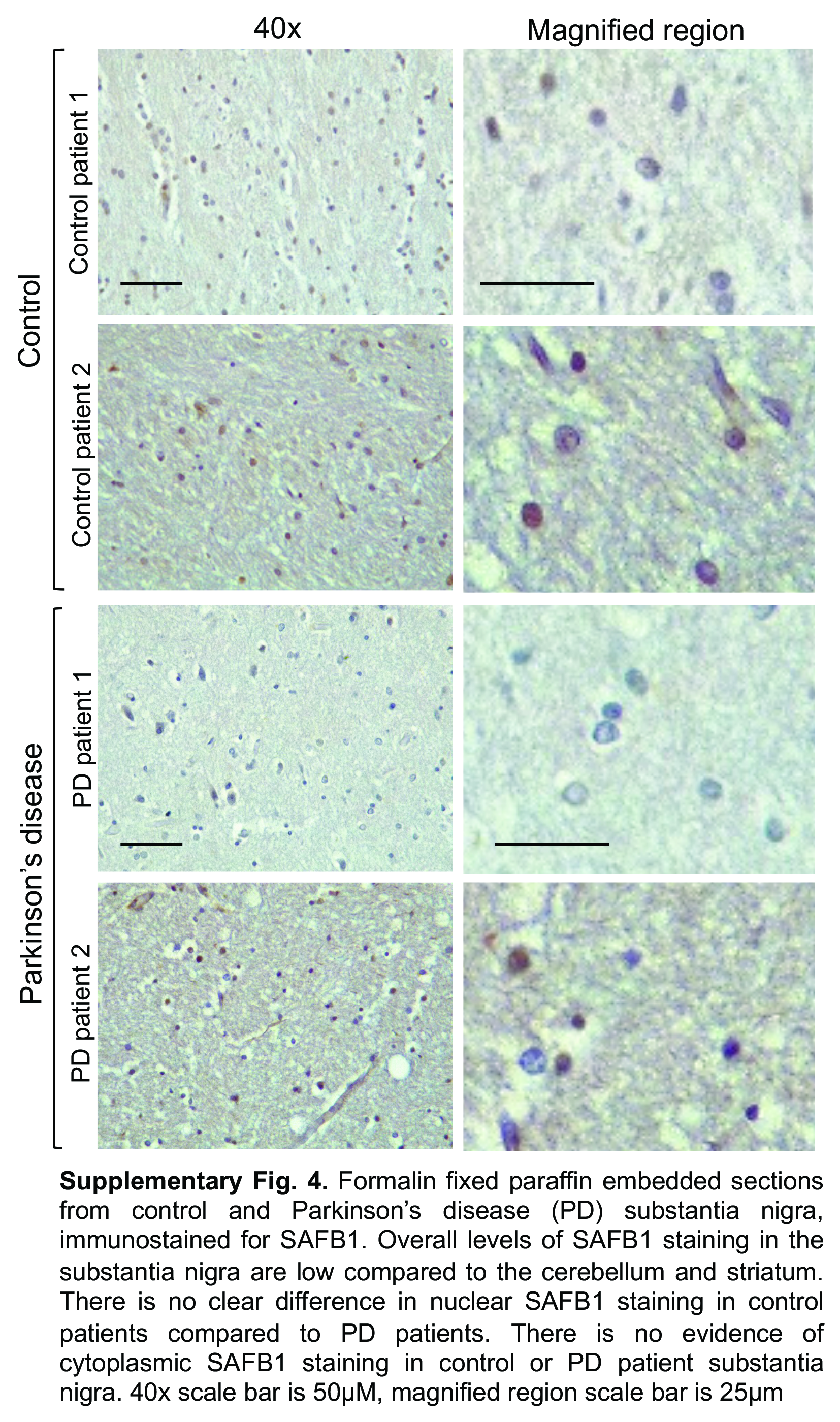

Supplement: Supplementary file 4 — Figure S4. Formalin fixed paraffin embedded sections from control and Parkinson’s disease (PD) substantia nigra, immunostained for SAFB1. Overall levels of SAFB1 staining in the substantia nigra are low compared to the cerebellum and striatum. There is no clear difference in nuclear SAFB1 staining in control patients compared to PD patients. There is no evidence of cytoplasmic SAFB1 staining in control or PD patient substantia nigra. 40× scale bar is 50 μM, magnified region scale bar is 25 μm. [file BPA-30-1041-s001.tif]
