# Supplementary material for: Abnormal scaffold attachment factor 1 expression and localization in spinocerebellar ataxias and Huntington’s chorea
Source: Brain Pathol. 2020 Jul 13;30(6):1041–55. doi: 10.1111/bpa.12872 (PMC8018166; doi:10.1111/bpa.12872)
Supplement: Supplementary file 5 — Table S1. Spinocerebellar ataxia (SCA) patient diagnosis and pathology data. [file BPA-30-1041-s002.docx]

|  |  |  | **Cerebellar neuropathological findings** | | | |
| --- | --- | --- | --- | --- | --- | --- |
| **Case**  **number​** | **Diagnosis​** | **Genetic diagnosis** | **Purkinje cells​** | **Dentate nucleus** | **​Granule cells** | **Polyglutamine positivity?** |
| **5** | Spinocerebellar ataxia type 1 | Confirmed SCA1 | Depleted, with shrinkage of remaining cells | Atrophy/shrinkage of neurons | Reduction in density | Staining not carried out |
| **​6** | Autosomal dominant spinocerebellar ataxia | Family history | Severe loss, with severe shrinkage of remaining cells. No torpedoes. | Some loss and shrinkage of neurons | Reduction in density | Staining not carried out |
| **7** | Spinocerebellar ataxia | Not available | Depleted with empty baskets. No torpedoes | Atrophy/shrinkage of neurons | Reduction in density | Staining not carried out |
| **8** | Spinocerebellar ataxia | Not available | Depleted, with heterotypic PCs in granule cell layer. No P62 positivity. Polyglutamine positivity in surviving PCs. | Atrophy/shrinkage of neurons | P62 positive inclusions | Strong polyglutamine staining in surviving PCs, strongly suggestive of polyQ SCA |
| **9** | Spinocerebellar ataxia | Not available | Severe loss, with severe shrinkage of remaining cells. | Some loss and shrinkage of neurons | Reduction in density | Staining not carried out |

Supplemental Table 1. Spinocerebellar ataxia (SCA) patient diagnosis and pathology data.
