# Supplementary material for: Abnormal scaffold attachment factor 1 expression and localization in spinocerebellar ataxias and Huntington’s chorea
Source: Brain Pathol. 2020 Jul 13;30(6):1041–55. doi: 10.1111/bpa.12872 (PMC8018166; doi:10.1111/bpa.12872)
Supplement: Supplementary file 6 — Table S2. Purkinje and dentate neurons counted and number of cells positive for nuclear and/or cytoplasmic SAFB1 staining. [file BPA-30-1041-s003.docx]

| **Case** | **Diagnosis** | **PCs counted** | **Nuclear SAFB1** | **Cytoplasmic SAFB1** |
| --- | --- | --- | --- | --- |
| 1 | Control | 326 | 226 | 3 |
| 2 | Control | 315 | 262 | 2 |
| 3 | Control | 321 | 182 | 10 |
| 4 | Control | 310 | 247 | 3 |
|  | **Total** | **1272** | **917** | **18** |
| 5 | SCA | 242 | 241 | 137 |
| 6 | SCA | 20 | 18 | 16 |
| 7 | SCA | 39 | 30 | 12 |
| 7 | SCA | 29 | 20 | 4 |
| 9 | SCA | 22 | 11 | 11 |
|  | **Total** | **352** | **320** | **180** |
| MS 1 | MS | 316 | 298 | 17 |
| MS 2 | MS | 417 | 404 | 7 |
| MS 3 | MS | 371 | 192 | 38 |
| MS 4 | MS | 208 | 192 | 6 |
|  | **Total** | **1312** | **1086** | **68** |

| **Case** | **Diagnosis** | **DN neurons counted** | **Nuclear SAFB1** | **Cytoplasmic SAFB1** |
| --- | --- | --- | --- | --- |
| 1 | Control | 143 | 21 | 1 |
| 2 | Control | 133 | 27 | 1 |
| 3 | Control | 126 | 17 | 4 |
| 4 | Control | 148 | 10 | 7 |
|  | **Total** | **550** | **75** | **13** |
| 5 | SCA | 112 | 81 | 76 |
| 6 | SCA | 135 | 90 | 46 |
| 7 | SCA | 148 | 39 | 48 |
| 8 | SCA | 181 | 135 | 155 |
| 9 | SCA | 267 | 216 | 233 |
|  | **Total** | **843** | **561** | **558** |

Supplemental Table 2. Purkinje and Dentate neurons counted and number of cells positive for Nuclear and/or cytoplasmic SAFB1 staining
